# Expression of Autophagy-Related Proteins According to Androgen Receptor and HER-2 Status in Estrogen Receptor-Negative Breast Cancer

**DOI:** 10.1371/journal.pone.0105666

**Published:** 2014-08-20

**Authors:** Ji-Ye Kim, Woo Hee Jung, Ja Seung Koo

**Affiliations:** Department of Pathology, Severance Hospital, Brain Korea 21 PLUS Project for Medical Science, Yonsei University College of Medicine, Seoul, South Korea; II Università di Napoli, Italy

## Abstract

**Purpose:**

The purpose of this study was to investigate the expression of autophagy-related proteins in relation to androgen receptor (AR) status in estrogen receptor (ER)-negative breast cancers.

**Methods:**

We extracted 334 ER-negative breast cancer samples to construct tissue microarrays (TMAs), which were immunohistochemically stained for autophagy-related proteins (beclin-1, LC3A, LC3B, p62) and for AR and HER-2.

**Results:**

There were 127 AR-positive cases and 207 AR-negative cases, and 140 HER-2-positive cases and 194 HER-2 negative cases. The AR-negative group was associated with tumoral LC3A expression (*P*<0.001), while the AR-positive group was associated with tumoral BNIP3 expression (*P*<0.001). Tumoral LC3A was most highly expressed in the AR-negative and HER-2 negative group, while stromal LC3A showed the highest expression in the AR-negative and HER-2-positive group. Tumoral BNIP3 and stromal BNIP3 were highest in the AR-positive and HER-2-negative group. In the AR-positive and HER-2-negative group, stromal p62 positivity was an independent factor that was statistically significant in its association with shorter disease-free survival (DFS) (Hazard ratio: 10.21, 95% CI: 1.130–92.31, *P* = 0.039). Shorter DFS was associated with tumoral LC3A positivity (Hazard ratio: 10.28, 95% CI: 2.068–51.19, *P* = 0.004) in the AR-negative and HER-2-positive group.

**Conclusion:**

In ER-negative breast cancers, AR status was associated with expression of different types of autophagy-related proteins. Tumoral LC3A was most highly expressed in AR-negative breast cancers, while tumor BNIP3 was highest in AR-positive breast cancers.

## Introduction

Autophagy is defined as the lysosomal degradation of cellular components within cells. There are three types of autophagy: microautophagy, chaperone-mediated autophagy and macroautophagy, which is the most commonly employed type. Autophagy removes dysfunctional or damaged cellular components while recycling cellular components that can be re-used, thereby playing an important homeostatic role within the cell [1]–[4]. Autophagy-related proteins that are used as markers to evaluate activation levels of autophagy include: beclin-1 [5]–[8], a protein known to participate in the nucleation process; LC3A [9]–[11], a protein that participates in the elongation process and thereby forms autophagosomes; P62, a scaffolding protein that transfers ubiquitinated protein to autophagosomes; and BNIP3, which plays a central role in mitophagy, the autophagy process within mitochondria. However, autophagy is not limited to normal cells; it is also reported to play a significant role in cancer cells. In general, the cancer cell adopts specialized metabolic processes through angiogenesis and/or aerobic glycolysis in their usual harsh hypoxic and nutrient-deficient environment. However, in particularly highly aggressive malignant tumors, where stresses are higher for metabolic demand, these specialized metabolic pathways may not be sufficient, so some tumors may adopt an alternative metabolic pathway of autophagy [12], [13]. In such cases, autophagy works to recycle cytoplasmic components to supply extra energy to the cell. Therefore, the autophagy process should be closely associated with metabolism in cancer progression and survival.

The development and natural history of breast cancer is significantly influenced by the status of steroidal hormones, such as those of estrogen. It is common practice to evaluate the status of estrogen receptor (ER) and progesterone receptor (PR) in order to treat and prognosticate breast cancer. In addition to ER/PR, androgen receptor (AR) is another steroidal hormone that influences and is associated with breast cancers, but the relationship is still not clearly understood. In general, androgen receptors are expressed in 70% of all breast cancers [14], with higher rates in apocrine and lobular types [15]. Previous studies have revealed that ER negativity is associated with glycolysis-related proteins such as Glut-1, CAIX and MCT-4 [16], [17]. Therefore, it has been suggested that ER-negative breast cancer has a higher metabolic activity than ER-positive breast cancer. Because the autophagy process should be closely associated with metabolism in cancer progression and survival, it is expected that autophagy activity is more elevated in ER-negative breast cancer than in ER-positive breast cancer. However, AR status and its association with autophagy-related proteins remain unexplored.

The purpose of this study was to investigate how autophagy-related proteins are expressed in relation to AR status in ER-negative breast cancers and to determine the corresponding clinical implications.

## Materials and Methods

### Patient Selection and Clinicopathologic Evaluation

Formalin-fixed paraffin-embedded tissue samples of patients diagnosed with invasive ductal carcinoma, no specific type, from January 2005 to December 2012 at Severance Hospital were included in this study. The study was approved by the Institutional Review Board (IRB) of Severance Hospital. IRB board waived the need for written informed consent. Those cases that had undergone pre-operative chemotherapy were excluded. Information on ER, AR and HER-2 status was collected from pathology reports. A cut-off value of 1% or more positively stained nuclei was used to define ER and AR positivity [18]. HER-2 staining was analyzed according to the American Society of Clinical Oncology (ASCO)/College of American Pathologists (CAP) guidelines using the following categories: 0 = no immunostaining; 1+ = weak incomplete membranous staining, less than 10% of tumor cells; 2+ = complete membranous staining, either uniform or weak in at least 10% of tumor cells; and 3+ = uniform intense membranous staining in at least 30% of tumor cells [19]. HER-2 immunostaining was considered positive when strong (3^+^) membranous staining was observed, whereas cases with 0 to 1^+^were regarded as negative. The cases showing 2+ HER-2 expression were evaluated for HER-2 amplification by fluorescent *in situ* hybridization (FISH).

All cases were retrospectively reviewed by a breast pathologist (Koo JS), in which histological evaluation was based on hematoxylin and eosin (H&E)–stained slides. The histological grade was assessed using the Nottingham grading system [20]. Tumor staging was based on the 7th American Joint Committee on Cancer (AJCC) criteria. Disease-free survival (DFS) was calculated from the date of the first curative surgery to the date of the first loco-regional or systemic relapse, or death without any type of relapse. Overall survival (OS) was estimated from the date of the first curative operation to the date of the last follow-up or death from any cause. Clinicopathologic parameters evaluated in each breast cancer included patient age at initial diagnosis, lymph node metastasis, tumor recurrence, distant metastasis, and patient survival.

### Tissue Microarray

After reviewing H&E–stained slides, the most suitable formalin-fixed, paraffin-embedded (FFPE) tumor tissue samples were retrospectively selected. The most representative tumor region on the FFPE sample was then marked and a 3-mm tissue core sample was extracted using a punch machine and planted onto a 6×5 recipient block. A total of 2 tissue cores were taken for all samples in this TMA construction.

### Immunohistochemistry

The antibodies used for immunohistochemistry in this study are shown in Table 1. Briefly, FFPE blocks were sectioned at a thickness of 3 um and then deparaffinized and rehydrated using xylene and alcohol solutions, respectively. Sections were then stained using the VentanaDiscoversy XT automated stainer (Ventana Medical System, Tucson, AZ, USA). Antigen retrieval was achieved by soaking sections in a CC1-buffered solution (Cell Conditioning 1; citrate buffer Ph 6.0, Ventana Medical System). The appropriate positive and negative controls were included together with the study sample for staining.

**Table 1:** Clone, dilution, and source of antibodies used.

Antibody	Clone	Dilution	Source
Autophagy related			
Beclin-1	Polyclonal	1∶100	Abcam, Cambridge, UK
LC3A	EP1528Y	1∶100	Abcam, Cambridge, UK
LC3B	Polyclonal	1∶100	Abcam, Cambridge, UK
p62	SQSTM1	1∶100	Abcam, Cambridge, UK
BNIP3	Ana40	1∶100	Abcam, Cambridge, UK

### Interpretation of Immunohistochemical Results

Interpretations of IHC stains were standardized as the proportion of stained cells multiplied by the intensity of the immunohistochemical staining. The proportion of stained cells was scored with a system ranging from 0 to 2, defined as follows: 0 represented a negative result, 1 represented a section in which less than 30% of cells were positively stained, and 2 represented a section in which more than 30% of cells were positively stained. Immunostaining intensity was scored with a system ranging from 0 to 3, defined as follows: 0 represented a negative result, 1 represented weak, 2 represented moderate, and 3 represented strong. The number obtained after the multiplication of stained cell proportion by immunostaining intensity resulted in the overall interpretation score: 0–1 was defined as negative, 2–6 as positive [21].

### Statistical Analysis

Data were statistically processed using SPSS for Windows version 12.0 (SPSS Inc., Chicago, IL). Student’s *t* test and Fisher’s exact test were used for continuous and categorical variables, respectively. To analyze data with multiple comparisons, a corrected *P*-value with application of the Bonferroni method for multiple comparisons was used. Statistical significance was assumed when *P*<0.05. Kaplan-Meier survival curves and log-rank statistics were employed to evaluate time to tumor metastasis and time to survival. Multivariate regression analysis was performed using a Cox proportional hazards model.

## Results

### Basal Characteristics of Patients According to the AR and HER-2 Status in ER-Negative Breast Cancer

There were 127 AR-positive cases, and 140 HER-2-positive cases. After dividing samples into four groups based on AR and HER-2 status, there were 53 cases in the AR (+)/HER-2 (–) group, 74 cases in the AR (+)/HER-2 (+) group, 66 cases in the AR (–)/HER-2 (+) group and 141 cases in the AR (–)/HER-2 (–) group. When ER negative cancer was divided into groups according to AR and HER-2 status, these groups exhibited a noticeable difference in age at diagnosis and Ki-67 expression levels. AR negative group was associated with older age and higher Ki-67 LI(*P* = 0.003, and *P*<0.001, respectively). While within the AR negative group, HER-2positive group was shown to be associated with older age at diagnosis and HER-2 negative group with higher Ki-67 LI(*P* = 0.030, and *P* = 0.002, respectively, Table 2).

**Table 2:** Clinicopathologic characteristics according to the AR and HER-2 status in ER-negative breast cancer.

			AR-positive group, n = 127			AR-negative group, n = 207				
Parameters	Sub-category	TotalN = 334 (%)	HER-2−n = 53 (%)	HER-2+n = 74 (%)	*P*-value	HER-2+n = 66 (%)	HER-2−n = 141 (%)	*P*-value	*P*-value*	*P*-value†
Age (years)					0.936			**0.030**	**0.001**	**0.003**
	≤35	34 (10.2)	2 (3.8)	3 (4.1)		4 (6.1)	25 (17.7)			
	>35	300 (89.8)	51 (96.2)	71 (95.6)		62 (93.9)	116 (82.3)			
Histologic grade					0.729			0.074	0.078	0.067
	I/II	136 (40.7)	26 (49.1)	34 (45.9)		30 (45.5)	46 (32.6)			
	III	198 (59.3)	27 (50.9)	40 (54.1)		36 (54.5)	95 (67.4)			
T stage					0.619			0.089	0.126	0.115
	T1	160 (47.9)	27 (50.9)	41 (55.4)		35 (53.0)	57 (40.4)			
	T2/T3	174 (52.1)	26 (49.1)	33 (44.6)		31 (47.0)	84 (59.6)			
Lymph node metastasis					0.839			0.354	0.594	0.373
	No	245 (73.4)	40 (75.5)	57 (77.0)		50 (75.8)	98 (69.5)			
	Yes	89 (26.6)	13 (24.5)	17 (23.0)		16 (24.2)	43 (30.5)			
Tumor recurrence					0.801			0.562	0.843	0.600
	No	295 (88.3)	48 (90.6)	66 (89.2)		59 (89.4)	122 (86.5)			
	Yes	39 (11.7)	5 (9.4)	8 (10.8)		7 (10.6)	19 (13.5)			
Patient death					0.950			0.442	0.242	0.080
	No	303 (90.7)	50 (94.3)	70 (94.6)		60 (90.9)	123 (87.2)			
	Yes	31 (9.3)	3 (5.7)	4 (5.4)		6 (9.1)	18 (12.8)			
Ki-67 LI (%)					0.294			**0.002**	**<0.001**	**<0.001**
	≤14	79 (23.7)	22 (41.5)	24 (32.4)		18 (27.3)	15 (10.6)			
	>14	31 (58.5)	31 (58.5)	50 (67.6)		48 (72.7)	126 (89.4)			

*P-value was from comparison among 4 groups.

† *P*-value was from comparison between AR+ and AR– groups by Fisher exact test.

### Expression of Autophagy-Related Proteins According to the AR and HER-2 Status in ER-Negative Breast Cancer

Among autophagy-related proteins, LC3A, LC3B, BNIP3 exhibited cytoplasmic expression. In the case of beclin-1 and p62 proteins, these showed cytoplasmic and nuclear expression. Reports in the literature indicate that when these proteins exhibited nuclear expression, they were unrelated with autophagy activity. Therefore, we eliminated those with nuclear expression from our data and only counted those with cytoplasmic expression as positive [22], [23].

**Figure 1:**
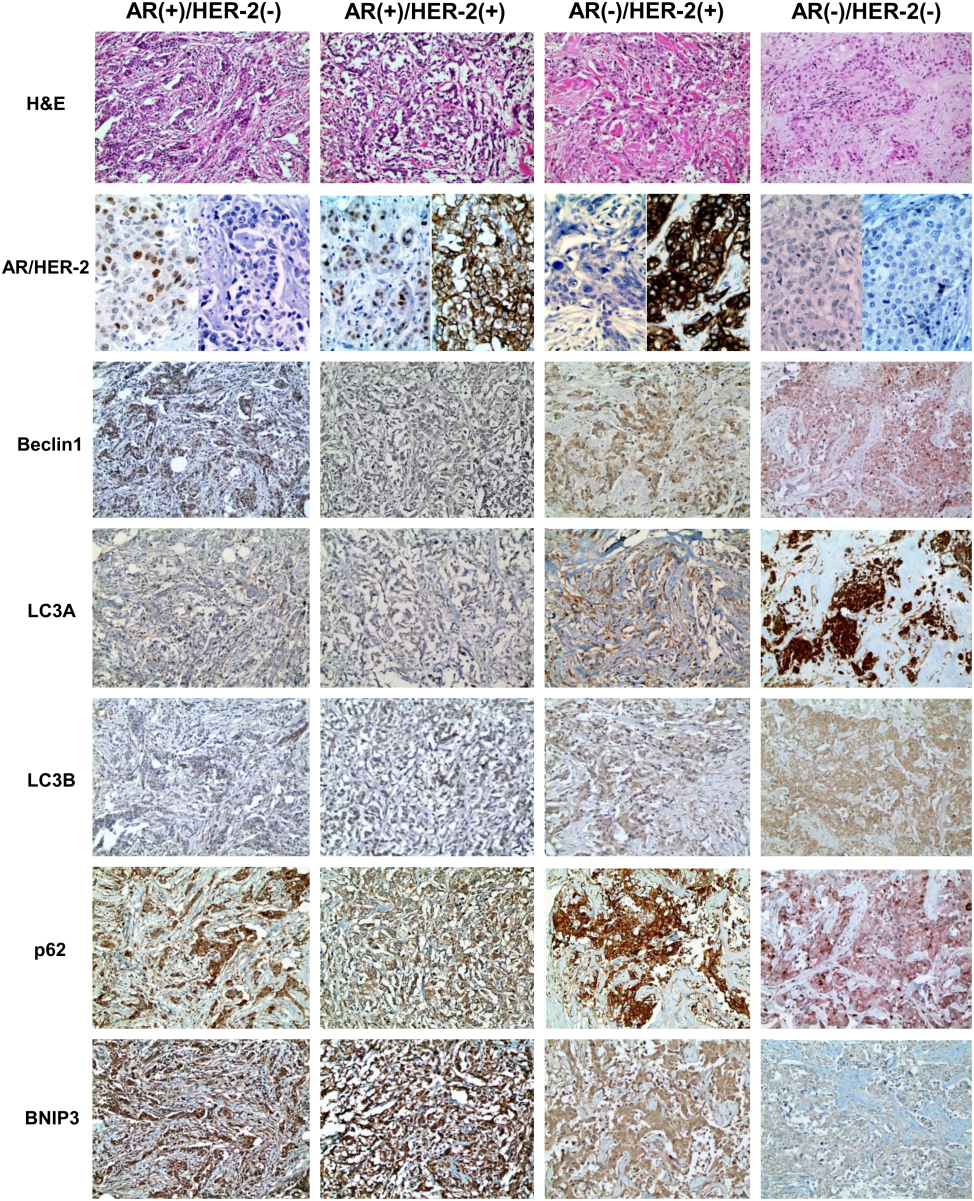
Expression of autophagy-related proteins according to AR and HER-2 status in ER-negative breast cancer. Tumoral LC3A expression was highest in the AR (–)/HER-2 (–) group, and lowest in the AR (+)/HER-2 (+) group. Stromal LC3A was highest in the AR (–)/HER-2 (+) group, and lowest in the AR (–)/HER-2 (–) group. Tumoral BNIP3 and stromal BNIP3 had the highest expression in the AR (+)/HER-2 (–) group and lowest in the AR (–)/HER-2 (–) group. Expression of Beclin-1, LC3B, and p62 is similar among 4 subgroups.

For expression of autophagy-related proteins according to AR and HER-2 status in ER-negative breast cancers, tumoral LC3A expression was highest in the AR (–)/HER-2 (–) group, and lowest in the AR (+)/HER-2 (+) group (*P*<0.001). Stromal LC3A was highest in the AR (–)/HER-2 (+) group, and lowest in the AR (–)/HER-2 (–) group (*P*<0.001). Tumoral BNIP3 and stromal BNIP3 had the highest expression in the AR (+)/HER-2 (–) group and lowest in the AR (–)/HER-2 (–) group (*P*<0.001, and *P* = 0.009, respectively, Figure 1).

For expression of autophagy-related proteins according to AR status, tumoral LC3A was expressed most highly in the AR-negative group (*P*<0.001), while tumoral BNIP3 was most highly expressed in the AR-positive group (*P*<0.001, and Table 3).

**Table 3:** Expression of metabolism-related proteins according to the AR and HER-2 status in ER-negative breast cancer.

Parameters	Total N = 334(%)	AR-positive group			AR-negative group			p-value*	p-value†
		HER-2−n = 53 (%)	HER-2+n = 74 (%)	p-value	HER-2+n = 66 (%)	HER-2−n = 141 (%)	p-value		
Beclin-1 (T)				1.000			0.074	0.185	0.260
Negative	184 (55.1)	31 (58.5)	44 (59.5)		41 (62.1)	68 (48.2)			
Positive	150 (44.9)	22 (41.5)	30 (40.5)		25 (37.9)	73 (51.8)			
LC3A (T)				0.067			**<0.001**	**<0.001**	**<0.001**
Negative	272 (81.4)	47 (88.7)	72 (97.3)		60 (90.9)	93 (66.0)			
Positive	62 (18.6)	6 (11.3)	2 (2.7)		6 (9.1)	48 (34.0)			
LC3A (S)				0.205			**<0.001**	**<0.001**	0.602
Negative	252 (75.4)	44 (83.0)	54 (73.0)		35 (53.0)	119 (84.4)			
Positive	82 (24.6)	9 (17.0)	20 (27.0)		31 (47.0)	22 (15.6)			
LC3B (T)				0.848			0.172	0.204	0.132
Negative	209 (62.9)	35 (66.0)	51 (68.9)		44 (66.7)	79 (56.0)			
Positive	125 (37.4)	18 (34.0)	23 (31.1)		22 (33.3)	62 (44.0)			
p62 (T)				0.260			0.061	0.153	0.814
Negative	116 (34.7)	21 (39.6)	22 (29.7)		17 (25.8)	56 (39.7)			
Positive	218 (65.3)	32 (60.4)	52 (70.3)		49 (74.2)	85 (60.3)			
p62 (S)				0.409			0.867	0.770	0.609
Negative	246 (73.9)	38 (71.7)	58 (78.4)		49 (74.2)	101 (72.1)			
Positive	87 (26.1)	15 (28.3)	16 (21.6)		17 (25.8)	39 (27.9)			
BNIP3 (T)				0.362			**<0.001**	**<0.001**	**<0.001**
Negative	257 (76.9)	28 (52.8)	46 (62.2)		48 (72.7)	135 (95.7)			
Positive	77 (23.1)	25 (47.2)	28 (37.8)		18 (27.3)	6 (4.3)			
BNIP3 (S)				0.088			**0.010**	**0.009**	0.328
Negative	304 (91.0)	44 (83.0)	69 (93.2)		56 (84.8)	135 (95.7)			
Positive	30 (9.0)	9 (17.0)	5 (6.8)		10 (15.2)	6 (4.3)			

*P-value was from comparison among 4 groups.

† *P*-value was from comparison between the AR+ and AR– group by Fisher exact test.

### Correlation between Clinicopathologic Parameters and Expression of Autophagy-Related Proteins in ER-Negative Breast Cancer

**Figure 2:**
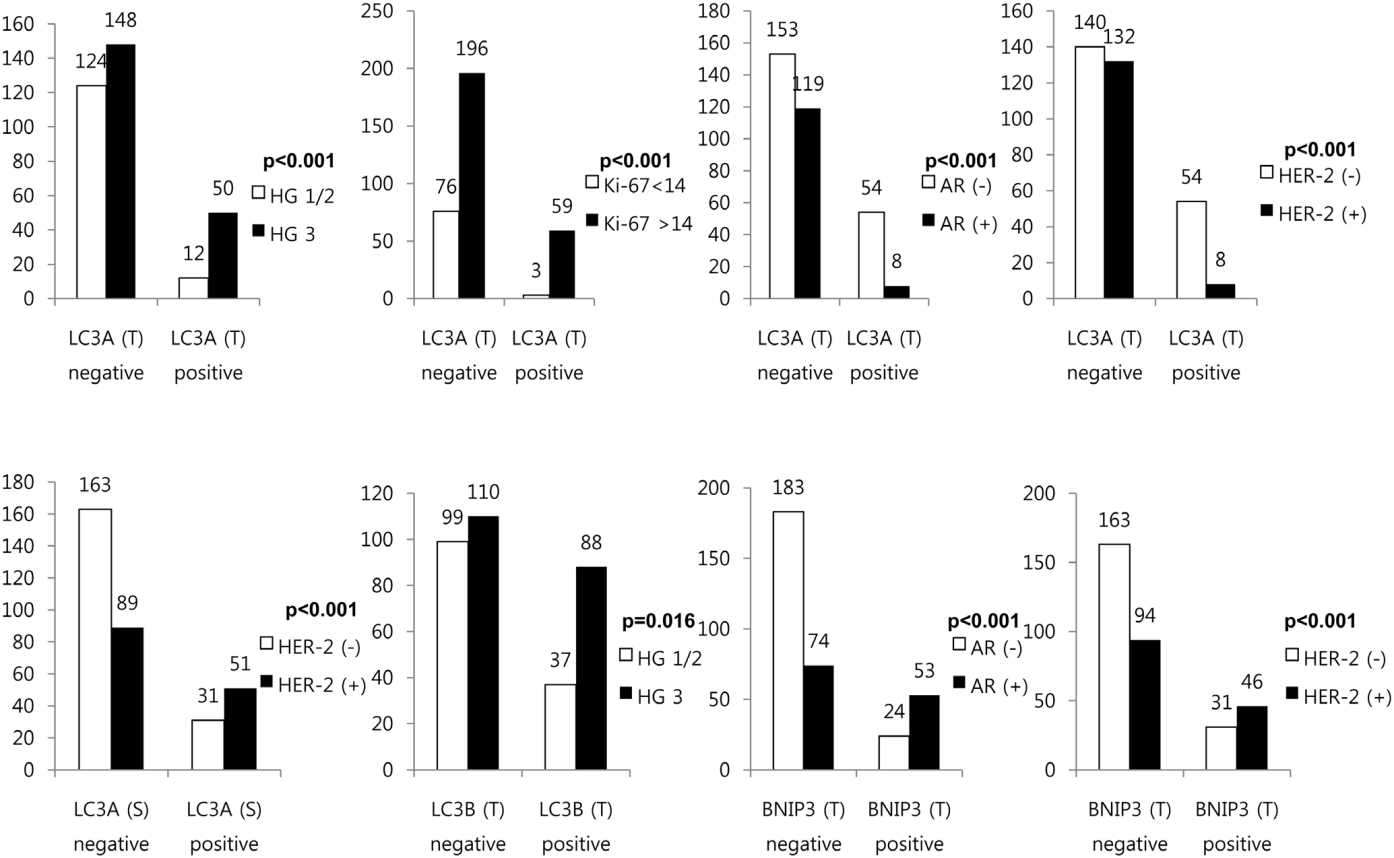
Correlation between clinicopathologic parameters and expression of autophagy-related proteins in ER-negative breast cancer.

Autophagy-related protein expression according to clinicopathologic parameters was analyzed in ER-negative breast cancer (Figure 2). Tumoral LC3A positivity was significantly associated with higher histologic grade (*P*<0.001), higher Ki-67 LI (*P*<0.001), AR negativity (*P*<0.001), and HER-2 negativity (*P*<0.001), while stromal LC3A positivity showed a significant relationship with HER-2 positivity (*P*<0.001). Tumoral LC3B positivity was associated with higher histologic grade (*P* = 0.016), while tumoral BNIP3 positivity was significantly associated with AR positivity (*P*<0.001) and HER-2 positivity (*P*<0.001).

**Figure 3:**
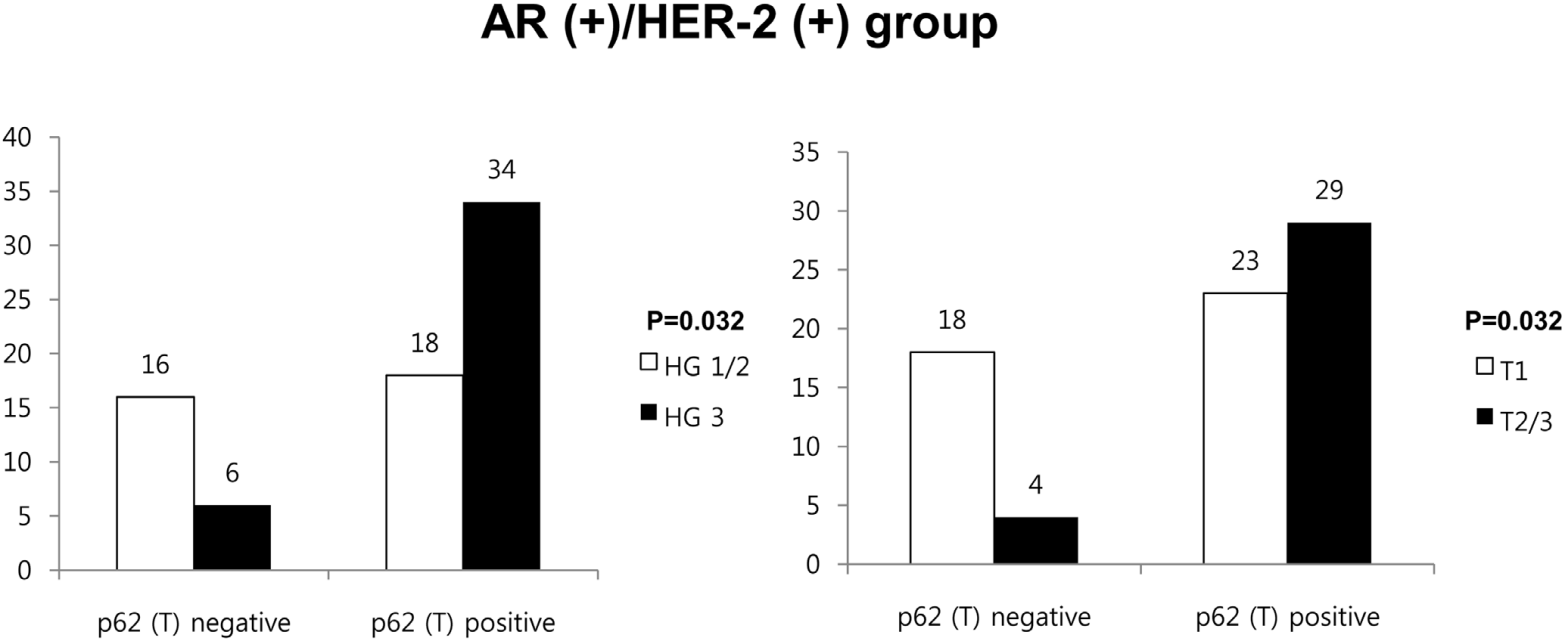
Correlation between clinicopathologic parameters and expression of autophagy-related proteins in the AR (+)/HER-2 (+) group.

In each of the four groups defined by AR and HER-2 status, we analyzed the autophagy-related protein expression according to clinicopathologic parameters. The AR (+)/HER-2 (+) group was the only group that showed a significant association between tumoral p62 positive and higher histologic grade (*P* = 0.032) as well as higher T stage (*P* = 0.032, Figure 3).

**Figure 4:**
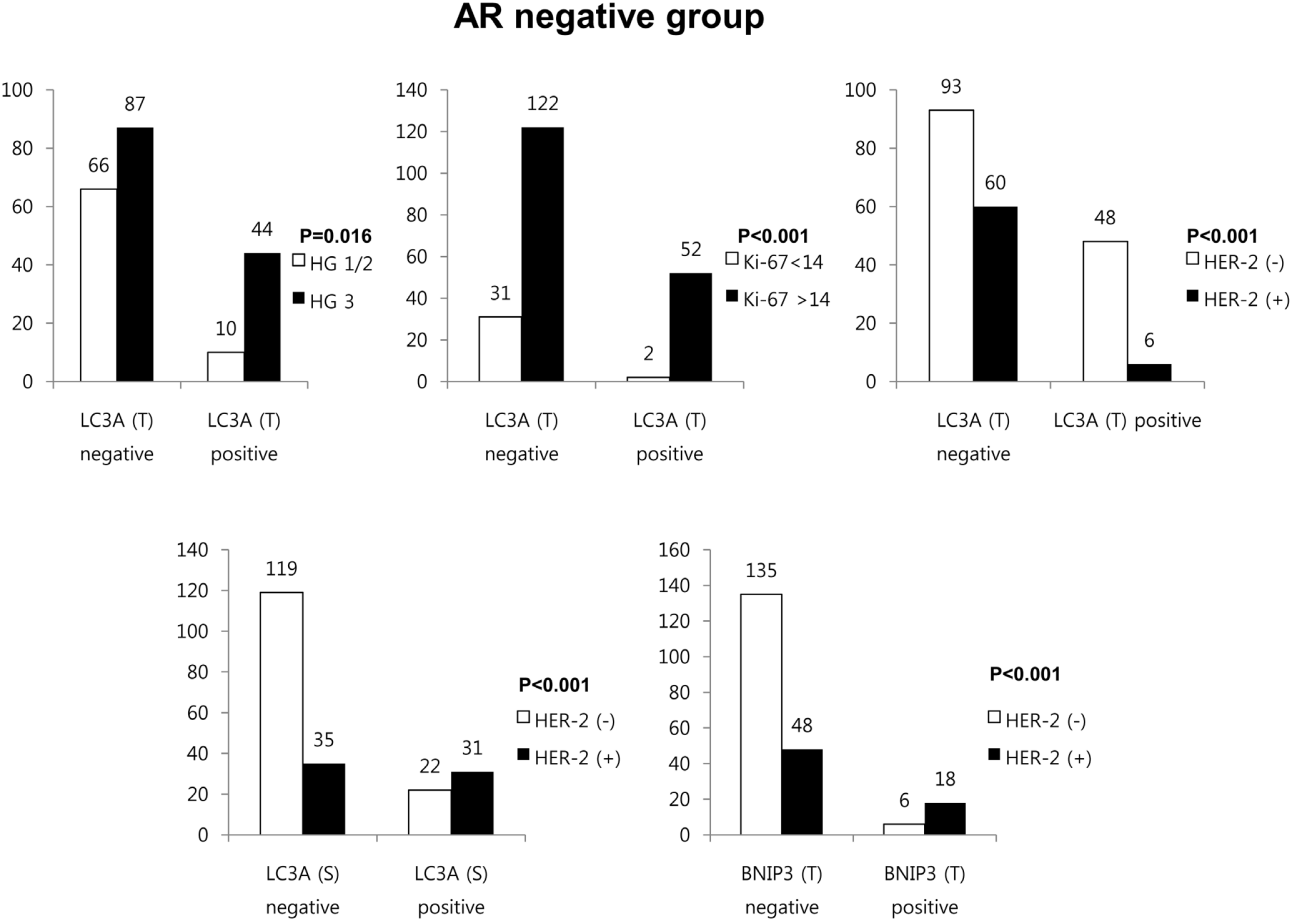
Correlation between clinicopathologic parameters and expression of autophagy-related proteins in the AR-negative group.

In each of the groups, autophagy-related protein expression with clinicopathologic parameters was assessed according to AR status (Figure 4). Within the AR-negative group, tumoral LC3A positivity was associated with higher histologic grade (*P* = 0.016), higher Ki-67 LI (*P* = 0.001), AR negativity (*P*<0.001) and HER-2 negativity (*P*<0.001). Stromal LC3A positivity was associated with HER-2 positivity (*P*<0.001) and tumoral BNIP3 positivity was associated with HER-2 positivity (*P*<0.001).

### Impact of Expression of Autophagy-Related Proteins on Prognosis

**Figure 5:**
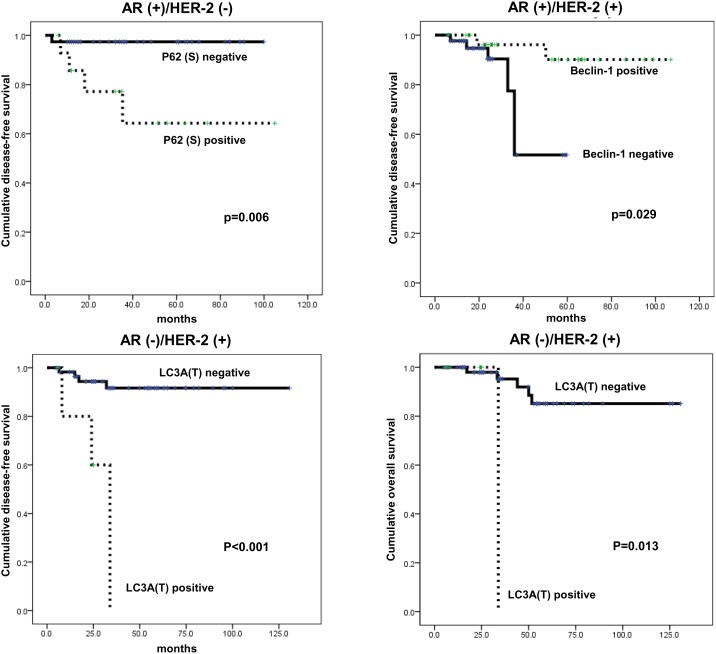
Impact of expression of autophagy-related proteins on prognosis according AR and HER-2 status in ER-negative breast cancer.

According to univariate analysis of autophagy-related protein expression with prognosis, no factors were significantly associated with prognosis (Table 4). However, amongst the four groups divided based on AR and HER-2 status, the AR (+)/HER-2 (–) group showed shorter disease-free survival (DFS) in association with stromal p62 positivity (*P* = 0.006), while in the AR (+)/HER-2 (+) group, shorter DFS was associated with tumoral beclin-1 negativity (*P* = 0.029). In the AR (–)/HER-2 (+) group, shorter DFS and shorter overall survival were significantly associated with tumoral LC3A positivity (*P*<0.001 and *P* = 0.013, respectively, Figure 5).

**Table 4:** Univariate analysis by log-rank test of the impact of metabolism-related protein expression in estrogen receptor-negative breast cancer on disease-free survival and overall survival times.

		Disease-free survival (months)		Overall survival (months)	
Parameters		95% CI	*P*-value	95% CI	*P*-value
Beclin-1 (T)			0.295		0.917
	Negative	114 (106–123)		124 (117–131)	
	Positive	97 (92–101)		119 (113–125)	
LC3A (T)			0.596		0.314
	Negative	117 (110–125)		123 (116–129)	
	Positive	111 (100–122)		129 (120–137)	
LC3A (S)			0.537		0.796
	Negative	117 (109–124)		125 (119–130)	
	Positive	75 (70–80)		76 (72–80)	
LC3B (T)			0.325		0.432
	Negative	114 (105–123)		121 (114–129)	
	Positive	116 (108–124)		121 (115–127)	
p62 (T)			0.907		0.289
	Negative	112 (99–125)		117 (106–128)	
	Positive	117 (111–124)		126 (120–131)	
p62 (S)			0.850		0.616
	Negative	118 (111–125)		126 (121–132)	
	Positive	91 (83–99)		111 (100–122)	
BNIP3 (T)			0.750		n/a
	Negative	117 (110–124)		n/a	
	Positive	90 (83–97)		n/a	
BNIP3 (S)			0.570		n/a
	Negative	117 (110–124)		n/a	
	Positive	93 (84–102)		n/a	

Multivariate Cox analysis revealed that in the AR (+)/HER-2 (–) group, stromal p62 positivity was an independent factor significantly associated with shorter DFS (Hazard ratio: 10.21, 95% CI: 1.130–92.31, *P* = 0.039, Table 5), while in the AR (–)/HER-2 (+) group, tumoral LC3A positivity was an independent factor significantly associated with shorter DFS (Hazard ratio: 10.28, 95% CI: 2.068–51.19, *P* = 0.004, Table 6).

**Table 5:** Multivariate analysis of DFS and OS in the AR (+)/HER-2 (–) group.

		Disease-free survival			Overall survival		
Included parameters		Hazard ratio	95% CI	*P*-value	Hazard ratio	95% CI	*P*-value
T stage				0.426			n/a
	T1 versus T2–3	2.693	0.235–30.85		n/a	n/a	
N stage				0.708			n/a
	N0 versus N1–3	1.409	0.234–8.491		n/a	n/a	
Histologic grade				0.608			n/a
	I/II versus III	1.895	0.165–21.71		n/a	n/a	
p62 (S)				**0.039**			n/a
	Negative vs positive	10.21	1.130–92.31		n/a	n/a	

**Table 6:** Multivariate analysis of DFS and OS in the AR (–)/HER-2 (+) group.

		Disease-free survival			Overall survival		
Included parameters		Hazard ratio	95% CI	*P*-value	Hazard ratio	95% CI	*P*-value
T stage				0.159			0.820
	T1 vs T2–3	4.749	0.542–41.59		1.227	0.211–7.149	
N stage				0.950			0.557
	N0 vs N1–3	1.054	0.207–5.370		1.690	0.293–9.736	
Histologic grade				0.773			0.776
	I/II vs III	0.794	0.167–3.789		0.774	0.132–4.522	
LC3A (T)				**0.004**			0.086
	Negative vs positive	10.28	2.068–51.19		10.52	0.718–154.3	

## Discussion

In the current study, expression of autophagy-related proteins according to AR status in ER-negative breast cancer was evaluated. Tumoral LC3A expression was highest in AR-negative breast cancer, while tumoral BNIP3 was highest in AR-positive breast cancer. In previous studies on invasive breast cancers, expression of autophagy-related proteins such as LC3A, LC3B, and beclin-1 was shown to be associated with ER negativity and PR negativity [24]. Therefore, it was suggested that hormone receptor-negative breast cancers were more closely associated with autophagy activity, and for ER-negative breast cancers, particularly AR-negative breast cancers, there was an association with increased autophagy activity. Although there are no studies that have explored the relationship between AR and autophagy in breast cancer, there have been studies of prostate cancer tumors, which are most representative of AR-positive tumors. While there are reports that AR positivity promotes autophagy in prostate cancer [25], there are reports of AR positivity with lower levels of autophagy activity [26], [27]. The current study revealed that there was an association between AR negativity and LC3A expression in which was that of an inverse relationship. This inverse relationship can be explained through an adapted interpretation of what has been already reported in prostate cancer studies. One study concluded that endoplasmic reticulum chaperone glucose-regulated protein 78/BiP(Grp 78/Bip) is upregulated by AR, which ultimately results in autophagy inhibition [26]. Another study demonstrated that AR increases p62 expression, which in turn inhibits autophagy [28]. Regardless of whether AR promotes or inhibits autophagy in prostate cancer, there is a consistent finding among these different studies that AR does play a role in cancer cell growth. Further *in vitro* cellular studies of AR and autophagy in breast cancer are required.

Our study is unique because this is the first in literature to analyze the relationship between BNIP3 and AR. Previous studies have done so far as to observe an overexpression of BNIP3 in breast cancers [29]. The relationship between BNIP3 and AR expressions would be explained through the mechanism of autocrine loop of tyrosine kinase receptor/phosphatidylinositol 3′-kinase/protein kinase B. In a AR positive prostate cancer cell study, androgen had activated this tyrosine kinase receptor/phosphatidylinositol 3′-kinase/protein kinase B which increases HIF-1α and HIF-α regulated gene expression [30]. BNIP3 happens to be one such HIF-α regulated gene [31].

In the current study of breast cancers, BNIP3 was highly expressed in the AR-positive group, which corresponded with a previous report of a significant association between BNIP3 expression and AR in prostate cancers [32]. The proposed mechanism for these findings is as follows; in prostate cancer, androgen is bound to AR, which activates HIF-1α through a cascade of several protein kinase systems, continuously increasing expression of the HIF-1α-related gene, one of which is BNIP3. BNIP3 increases mitophagy and prevents oxidative phosphorylation in mitochondria [33], [34]. Therefore, metabolism of tumor cells is shifted from mitochondrial oxidative phosphorylation to oxidative glycolysis, known as the Warburg effect [35]. Although it remains unclear if similar mechanisms are at work between AR and BNIP3 in breast cancer, as shown with prostate cancer, this study demonstrates that the AR-positive group has higher expression of BNIP3 in ER-negative breast cancer, suggesting possible causal mitochondrial dysfunction within the AR-positive group in ER-negative breast cancer.

Within the AR-negative group, there was a significant difference in LC3A and BNIP3 expression between the HER-2-positive and HER-2-negative groups. However, in the AR-positive group, there was no significant difference between HER-2-positive and HER-2-negative groups in autophagy-related protein expression. The ER (–)/AR (–)/HER-2 (+) group can be presumed to be mostly the HER-2 type, and the ER (–)/AR (–)/HER-2 (–) group is thought to be the basal-like/triple negative types. Because HER-2 type and basal-like/triple negative type are both distinct clinicopathologic entities [36], [37], differences in autophagy activity according to HER-2 status in the AR-negative group could be explained. The ER-negative and AR-positive group could be classified as molecular apocrine breast cancer (MABC) according to surrogate immunohistochemical markers in this study. In the literature, MABC is reportedly 20–50% HER-2 overexpressed/amplified [38], [39]. In the current study, 58.3% had HER-2 overexpression/amplification that was similar to the reported value. Although HER-2 status is an important biomarker in breast cancers, the current study revealed that there is no difference in the expression of autophagy-related proteins between the AR (+)/HER-2 (–) group and AR (+)/HER-2 (+) group, which is compatible with results from a previous study indicating that MABC does not exhibit differences in tumor characteristics according to HER-2 status [38].

Our study observed cytoplasmic expression of LC3A, LC3B and BNIP3 and nuclear expression in beclin-1 and p62. Previous reports noted that different expression patterns of LC3A had resulted in different biologic behaviors of tumors; in tumors with diffuse cytoplasmic or perinuclear LC3A expression had an association with ER and PR positivity, whereas those tumors with stone-like pattern of LC3A expression was associated with ER and PR negativity and a worse prognosis [10]. In the current study, there were no cases with stone-like pattern expression of LC3A. Instead, the majority exhibited cytoplasmic and/or perinuclear pattern. This may be due to difference in the antibody used from previous reports. However there are still other studies that support the observation that according to the type of tumor there may be no stone-like pattern expression of LC3A. All in all, LC3A seems to be an area that merits much study [40], [41]. In the case of p62 and beclin1, nuclear expression was observed in addition to cytoplasmic expression. This finding is in agreement with previous reports that observed both nuclear and cytoplasmic expression of p62 [42], [43]. This finding is attributed to the fact that p62 is a major component of the nuclear pore complex that functions as a nucleocytoplasmic transport thereby allowing it to exist both in nucleic and cytoplasmic compartments [22]. Beclin1 is also known to be expressed in both nucleic and cytoplasmic compartments [23]. Although there is no study dealing with the nuclear expression of beclin1, in one study on brain tumors, beclin1 tended to shift towards nucleic expression as the grade of the tumor worsens. This observation signified that beclin1 protein transports between both nucleic and cytoplasmic compartments, but more importantly this shift in expression implied the loss of becline1 gene function [23]. In summary, nucleic expression of beclin1 would imply a suspension of its role in autophagy regulation.

Although there was no association between autophagy-related protein expression and breast cancer prognosis in ER-negative breast cancer, in the AR (+)/HER-2 (–) group, stromal p62 positivity was an independent factor associated with shorter DFS, while in the AR (–)/HER-2 (+) group, tumoral LC3A positivity was an independent factor for shorter DFS. Previous studies have revealed that LC3A expression in ovarian clear cell carcinoma [44], non-small cell lung carcinoma [45], and colorectal adenocarcinoma [46] is associated with poor prognosis, providing a basis for the claim that increased autophagy associates with poor prognosis. With this in mind, the reason that LC3A was associated with poor prognosis only in the AR (–)/HER-2 (+) group is a question that requires further study. Also, it was stromal p62 expression that was associated with prognosis rather than tumoral p62 expression. Expression of p62, LC3A, LC3B, and BNIP3 in stromal cells in breast cancer was reported in a previous study [24]. The association between stromal p62 expression and poor prognosis may be explained by the reverse Warburg effect theory. The reverse Warburg effect is a theory that proposes a metabolic interaction between tumor cells and stromal cells in breast cancer, where the reactive oxygen species produced from tumor cells result in glycolysis, mitochondrial dysfunction, and increased autophagy in stromal cells. In addition, the lactate produced from stromal cell glycolysis is in turn utilized by the tumors in oxidative phosphorylation to produce ATP [47]–[51]. Therefore, breast cancers that produce energy by the reverse Warburg effect have an advantage in tumor growth and maintenance. If the AR (+)/HER-2 (–) group, in which stromal cells express p62, is presumed to produce energy by the reverse Warburg effect, it may be then associated with poor prognosis. The stromal cell that shows increased autophagy activity is called the cancer-associated fibroblast according to the reverse Warburg effect theory, and it is characterized by caveolin-1 loss[49]. Caveolin-1 loss is reported to occur in 5–40% of all breast cancers [52]–[54], and therefore only a portion of breast cancers would be exhibiting the reverse Warburg effect.

In conclusion, there was a significant difference in autophagy-related protein expression according to AR status in ER-negative breast cancer. Tumoral LC3A expression was higher in AR-negative breast cancer, while tumoral BNIP3 was higher in AR-positive breast cancer.
